# Current status and future prospects of pretreatment for tobacco stalk lignocellulose

**DOI:** 10.3389/fbioe.2024.1465419

**Published:** 2024-08-14

**Authors:** Nianwu Hu, Xiongbin Liu, Shuoguo Wei, Jianwu Yao, Wanxia Wang, Ben Liu, Tianming Tang, Jungang Jiang, Lei Wang

**Affiliations:** ^1^ China Tobacco Hubei Industrial Co., Ltd., Wuhan, China; ^2^ Hubei Xinye Reconstituted Tobacco Development Co. Ltd., Wuhan, China; ^3^ Applied Technology Research of Reconstituted Tobacco Hubei Province Key Laboratory, Wuhan, China; ^4^ Hubei Provincial Key Laboratory of Green Materials for Light Industry, Hubei University of Technology, Wuhan, China

**Keywords:** tobacco stalk, lignocellulose, pretreatment, valorization, biorefinery

## Abstract

With the growing demand for sustainable development, tobacco stalks, as a resource-rich and low-cost renewable resource, hold the potential for producing high-value chemicals and materials within a circular economy. Due to the complex and unique structure of tobacco stalk biomass, traditional methods are ineffective in its utilization, making the pretreatment of tobacco stalk lignocellulose a crucial step in obtaining high-value products. This paper reviews recent advancements in various pretreatment technologies for tobacco stalk lignocellulosic biomass, including hydrothermal, steam explosion, acid, alkaline, organic solvent, ionic liquid, and deep eutectic solvent pretreatment. It emphasizes the impact and efficiency of these pretreatment methods on the conversion of tobacco stalk biomass and discusses the advantages and disadvantages of each technique. Finally, the paper forecasts future research directions in the pretreatment of tobacco stalk lignocellulose, providing new insights and methods for enhancing its efficient utilization.

## 1 Introduction

**FIGURE 1:**
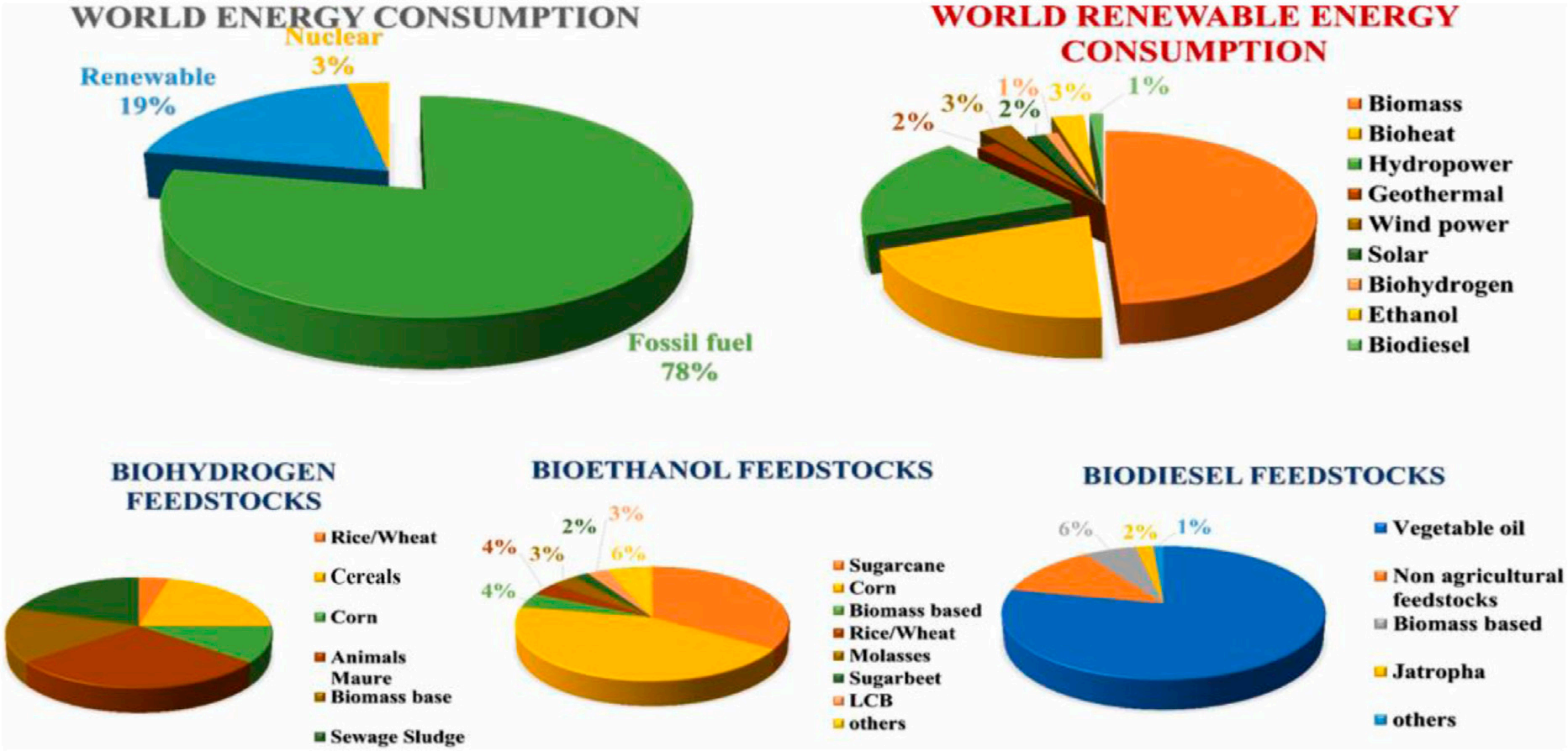
World energy consumption, world renewable energy consumption, production of biohydrogen, bioethanol, and biodiesel for feedstocks (Velvizhi et al., 2023).

With the rapid growth of the global population and the acceleration of industrialization, the massive consumption of traditional fossil energy and its associated environmental pollution have become increasingly prominent (Zimmerman et al., 2020; Ding et al., 2024). The excessive reliance on fossil fuels not only leads to a significant increase in greenhouse gas emissions but also exacerbates the severity of global climate change. Against this backdrop, the exploration and development of sustainable energy and material resources have become a pressing task for promoting sustainable socio-economic development (Figure 1) (Velvizhi et al., 2023). Biomass resources, as an important renewable energy source, not only boast abundant reserves but also can mitigate environmental pollution through their recycling, thereby achieving sustainable resource development (Queneau and Han, 2022; Tan and Tan, 2022). The advancement of biomass pretreatment and conversion technologies has enabled its transformation into a variety of high-value products such as biofuels, biobased chemicals, and biomaterials, further enhancing resource efficiency and environmental protection (Ding et al., 2024; Jiang et al., 2024; Singh et al., 2024).

Tobacco is an important non-food economic crop globally, extensively cultivated in countries such as China, India, Brazil, and the United States (Sun et al., 2019). However, a large amount of tobacco stalk by-products produced during the traditional cigarette manufacturing process is often discarded or used as low-value solid fuel due to the lack of effective economic utilization methods (Chen et al., 2019; Dallé et al., 2021; Santana et al., 2023). It is reported that in 2022, the production of tobacco stalks reached 5.8 million tons (Santana et al., 2024). However, these tobacco stalk wastes are not effectively utilized. In that case, it not only results in a tremendous waste of resources but also poses severe environmental impacts due to the high content of toxins such as nicotine, which increase the pollution load on soil and water sources (Lu and Lu, 2019). Moreover, the burning of tobacco stalks releases a large amount of CO_2_ and other harmful gases, further intensifying the greenhouse effect and air pollution problems (Zafeiridou et al., 2018). Therefore, developing effective tobacco straw reuse strategies to transform them into valuable renewable materials has become essential to addressing the aforementioned issues.

Tobacco stalks are mainly composed of cellulose, hemicellulose, and lignin, which endows them with high utilization potential (Cai et al., 2016; Sun et al., 2020; Yao et al., 2024). However, they also contain nicotine, addictive and toxic chemicals that significantly inhibit the activity of microorganisms and enzymes, negatively impacting the ecological environment and posing obstacles to the biotransformation process (Rogowska-van der Molen et al., 2023). Additionally, compared to other biomass resources, tobacco stalks have a more complex structure and stronger biomass recalcitrance (Chen et al., 2022). Therefore, developing effective pretreatment technologies to remove harmful components and break down their complex structure is crucial for realizing the resource utilization of tobacco stalks.

Unlike traditional biomass pretreatment, tobacco stalk pretreatment faces unique challenges due to the presence of toxic compounds such as nicotine (England et al., 2017). Conventional physical pretreatment methods, such as mechanical crushing or ultrasonic pretreatment, can disrupt the physical structure of tobacco stalks but fail to remove these toxic substances (Zhou and Tian, 2022). Thus, specific chemical pretreatment technologies must be employed to eliminate harmful components like nicotine from tobacco stalks effectively. Such pretreatment not only promotes the effective decomposition of biomass components but also increases the exposure of cellulose and hemicellulose, significantly enhancing the efficiency of subsequent biotransformation processes. This in-depth pretreatment method is crucial for realizing the high-value utilization of tobacco stalks.

This review starts with a review of the high-value utilization of tobacco stalks and critically explores the latest advancements in tobacco stalk pretreatment technologies. It discusses the advantages, disadvantages, technical limitations, and potential benefits of various emerging pretreatment technologies. Through the development of these technologies, tobacco stalks can not only serve as a source of bioenergy but also be used for producing various chemicals and materials, offering new approaches and pathways for addressing current energy and environmental challenges. This review aims to provide a new perspective for research on biomass refining based on tobacco stalks and offers theoretical and technical support for its comprehensive utilization in future developments.

## 2 Characteristics of tobacco stalk biomass

**FIGURE 2:**
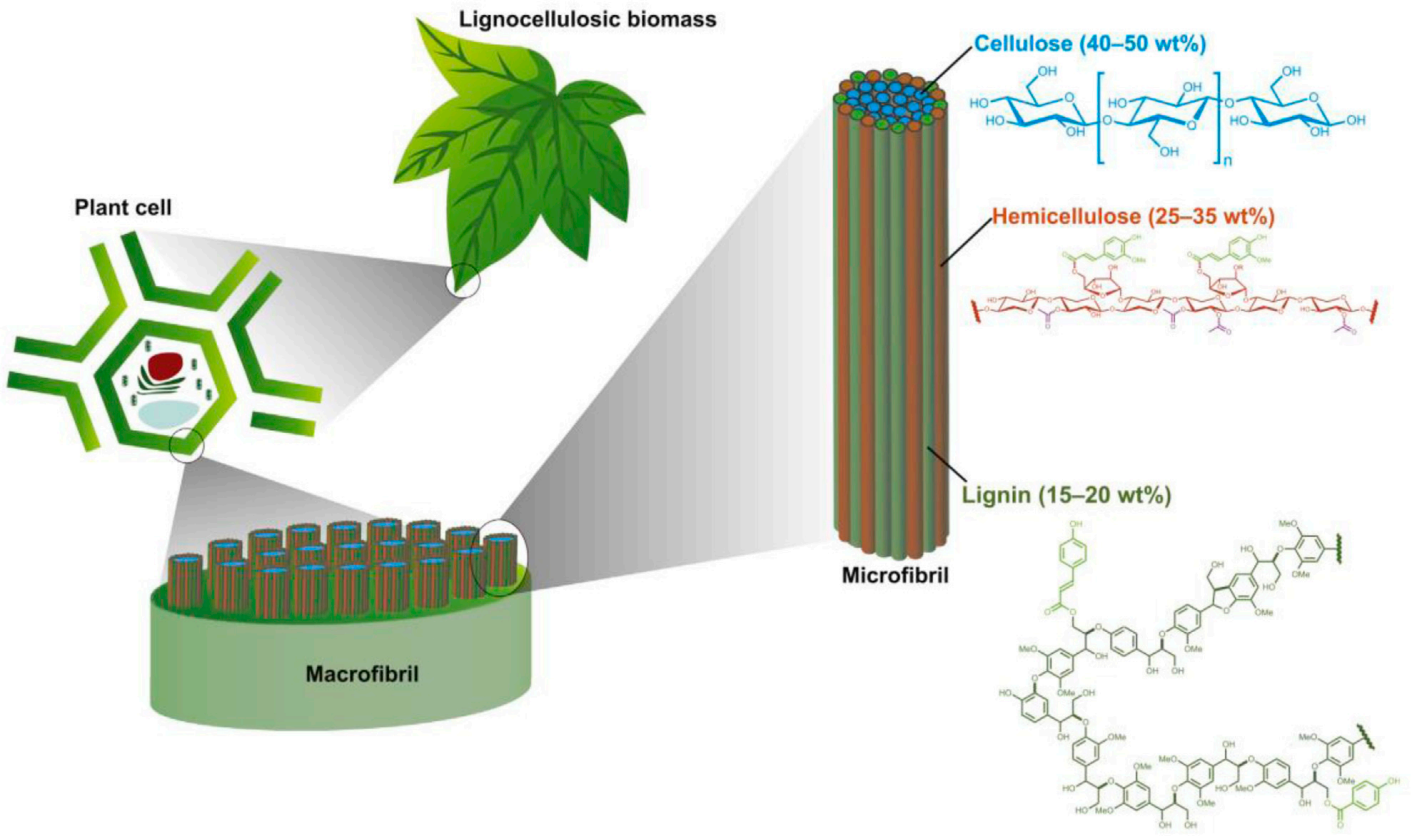
Overview of the structure of gramineous biomass (Bertella and Luterbacher, 2020).

Tobacco stalks, as a typical lignocellulosic biomass, are primarily composed of cellulose, hemicellulose, and lignin (Figure 2) (Sun et al., 2019; Bertella and Luterbacher, 2020; Sun et al., 2020). The complex arrangement and strong cross-linking of these components result in a robust lignocellulosic structure that is resistant to enzymatic and chemical degradation. The basic structural unit of cellulose is the D-glucose unit, which forms a linear polymer through β-(1,4)-glycosidic bonds, with a degree of polymerization ranging from 1,600 to 3,000 (Kaya et al., 2018). Cellulose exists in the form of microfibrils, where hydrogen bonds and van der Waals forces are the primary links connecting the crystalline and amorphous cellulose polymers (Tardy et al., 2021). Hemicellulose and lignin primarily cover these microfibrils. Non-covalent hydrogen bonds connect parts of the crystalline cellulose fibers, making them 3–30 times less degradable than the amorphous parts (Mali and Sherje, 2022). Although cellulase enzymes can hydrolyze the more accessible amorphous cellulose, they are less effective at decomposing the crystalline part. Hemicellulose is the second most common heterogeneous branched polymer in tobacco stalks (Rao et al., 2023). It comprises several components, including pentoses (D-xylose and L-arabinose) and hexoses (D-glucose, D-mannose, D-galactose, and L-rhamnose) (Deng et al., 2023; Santana et al., 2024). Compared to individual monosaccharide units, the degree of polymerization of hemicellulose is about 50–300 (Rao et al., 2023). The branched structure and the presence of acetyl groups result in hemicellulose lacking a crystalline structure. Moreover, due to its amorphous nature, hemicellulose is easily degradable. As a physical barrier, hemicellulose restricts the decomposition of cellulose by cellulase enzymes. The efficiency of cellulose hydrolysis can be enhanced by increasing enzyme loading and using steam hydrolysis or acid pretreatment methods to remove hemicellulose (Cheng et al., 2023a). Lignin is an amorphous polyphenolic polymer, and its structure is the most difficult to degrade (Shen et al., 2020; Cheng et al., 2023b). Tobacco stalk lignin typically consists of three types of methoxylated phenylpropanoid monomers (monolignols; namely, *p*-coumaric alcohol, coniferyl alcohol, and sinapyl alcohol) (Wang et al., 2022). The composition of lignin can vary based on the distribution ratio of different monomeric units across biomass sources. Lignin acts as a glue in the cell wall, surrounding the cellulose and hemicellulose fibers, providing mechanical strength and support for the formation of vascular tissues, facilitating nutrient transport, and enhancing resistance to microbial activity. Lignin is considered a key component that contributes to biomass recalcitrance because its phenolic structure occupies the spaces between the polysaccharides, reducing the accessibility of cellulase enzymes to cellulose. Furthermore, lignin-derived soluble compounds have also been found to cause enzyme deactivation.

Additionally, tobacco stalk biomass contains other substances such as nicotine, solanesol, pectin, proteins, amino acids, organic acids, and various sugars and ash rich in potassium, calcium, silicon, and magnesium (Akpinar et al., 2010; Huang et al., 2019). Unlike the lignocellulosic components, these elements not only determine the odor and color of the tobacco stalks but also play crucial roles in energy storage and protecting the plant from microbial damage. By understanding the structure and components of tobacco stalk biomass, we can better design and optimize pretreatment methods to enhance the utilization efficiency of tobacco stalks, facilitating their high-value applications in bioenergy, chemicals, and materials.

## 3 Role of pretreatment in lignocellulose valorization of tobacco stalk biomass

Tobacco stalk-based biorefining focuses on utilizing every fraction of the biomass to make the process economically feasible. Although tobacco stalks contain large amounts of fermentable sugars bound in cellulose and hemicellulose, these sugars cannot be directly utilized to synthesize value-added chemicals (Meng and Ragauskas, 2014). It is believed that without any pretreatment, a maximum of 20% of the theoretical yield of reducing sugars can be achieved through enzymatic hydrolysis (Gong et al., 2023). The remaining 80% loss is due to the recalcitrant nature of the biomass, which impedes its complete enzymatic hydrolysis.

**FIGURE 3:**
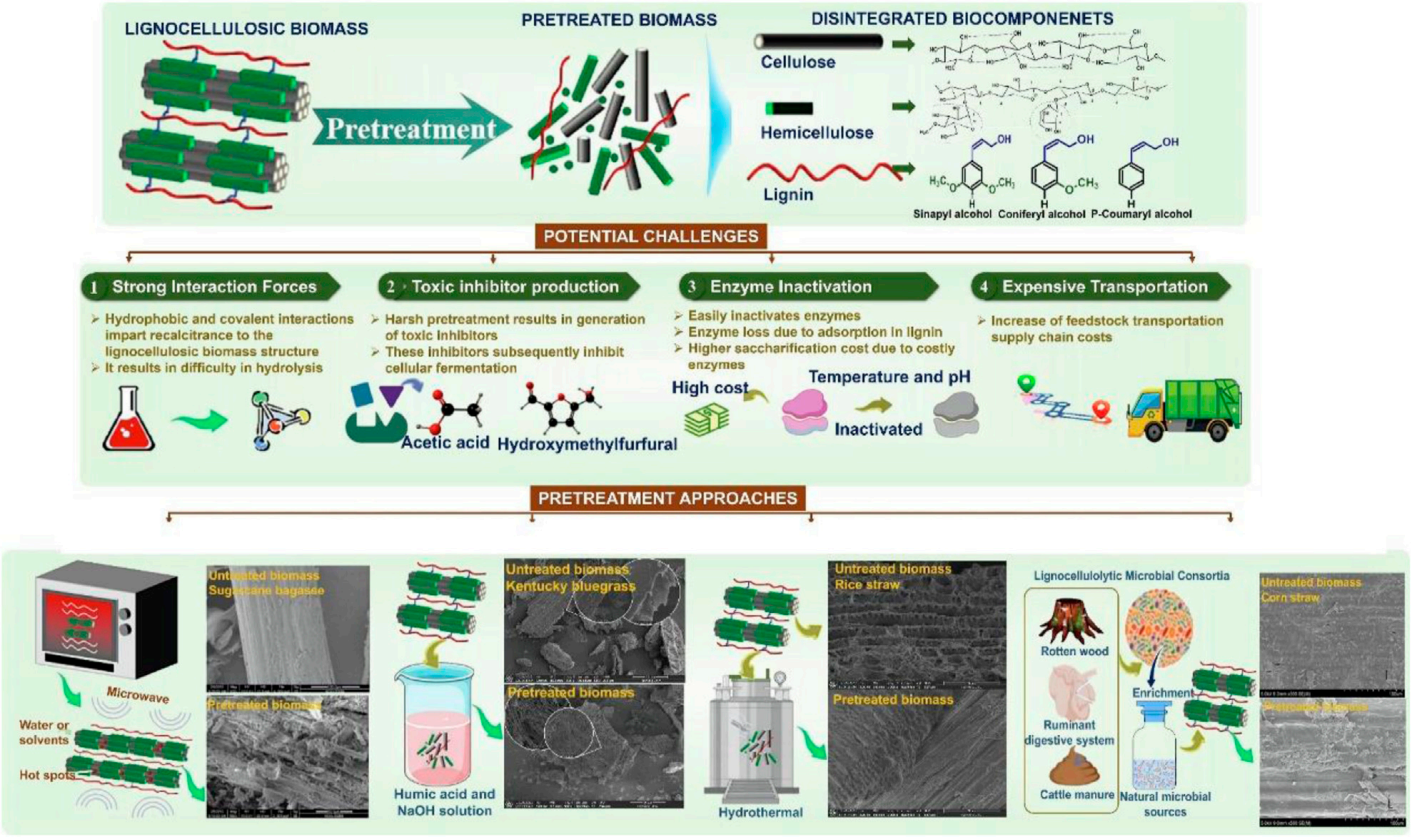
Defragmentation of lignocellulosic biomass, pretreatment challenges, and pretreatment approaches for its maximum valorization (Chakraborty et al., 2024).

Effective utilization of tobacco stalks follows a sequential process, starting with size reduction, followed by the most appropriate pretreatment to disintegrate the biomass, and ending with mild enzymatic hydrolysis to yield fermentable sugars (Figure 3) (Chakraborty et al., 2024). The role of pretreatment is to open up the complex structure of tobacco stalk biomass by breaking the inter- and intra-molecular bonds between lignin, cellulose, and hemicellulose, making cellulose more accessible to enzymatic attack (Mankar et al., 2021). Additionally, biomass pretreatment partially or completely solubilizes the lignin and hemicellulose fractions in the liquid medium. Eventually, all three fractions can be utilized for their respective functions. Different pretreatment methods act differently on biomass: some methods efficiently solubilize lignin, others completely hydrolyze the hemicellulose fraction, while others reduce the crystallinity and degree of polymerization of cellulose to enhance enzymatic conversion (Mankar et al., 2021). Therefore, different types of biomass may require different pretreatment methods according to their lignocellulosic composition, which can significantly increase pretreatment costs and decrease sugar yields.

Pretreatment of tobacco stalk biomass is the most expensive step in any biomass processing and may determine the fate of the overall biomass valorization process. Therefore, an ideal pretreatment method should have some common features, such as the ability to process large amounts of feedstocks, efficient separation of all fractions of biomass, substantial enhancement of enzymatic hydrolysis, low energy demands, low capital investment, ambient process conditions, and insignificant generation of fermentation inhibitors.

## 4 Chemical pretreatment techniques in tobacco stalk

Unlike other conventional lignocellulosic biomass, the pretreatment of tobacco stalk lignocellulose must address not only the barriers caused by the tightly bound structures of lignocellulosic and cell wall components (lignin, hemicellulose, and cellulose) to facilitate efficient enzymatic production of fermentable sugars but also the removal of toxic substances such as nicotine from the biomass (Sun et al., 2020). This ensures safe and high-value subsequent utilization. In most cases, pretreatment is an indispensable step despite being energy and cost-intensive. Without it, the yield of fermentable sugars from enzymatic hydrolysis would be too low to be viable for biorefineries. Pretreatment determines the enzymatic digestibility of lignocellulosic substrates and also affects downstream sugar fermentation due to the potential formation of inhibitors, by-products from lignin and hemicellulose, and wastewater treatment requirements, thereby impacting the overall economics of biorefining. This section will review the currently effective pretreatment technologies and evaluate their advantages, disadvantages, and potential application prospects (Table 1).

**TABLE 1:** Comparative overview of pretreatment techniques for tobacco stalk biomass.

Pretreatment method	Conditions (temperature, time, etc.)	Main products obtained
Hydrothermal Pretreatment	Water, 160°C–270°C, Pressure > 5 MPa, 20–40 min	Solubilized hemicellulose, high yield of fermentable sugars
Steam Explosion Pretreatment	Water steam, 160°C–270°C, Pressure > 5 MPa, 20–40 min	Hydrolyzed hemicellulose, high yield of fermentable sugars
Acid Pretreatment	Dilute acid, about 120°C, 90 min	High yield of fermentable sugars, lignin removal
Alkaline Pretreatment	Alkaline, 90°C, various times	Enhanced solubilization of lignin, high yield of fermentable sugars
Organosolv Pretreatment	Alcohol or other organic solvents, about 150°C–180°C, 60 min	Efficient lignin removal, high yield of fermentable sugars
Ionic Liquid Pretreatment	Imidazolium-based ionic liquids, 40°C–140°C, 1–2 h	Efficient separation and recovery of lignin, high yield of fermentable sugars
Deep Eutectic Solvents Pretreatment	Choline chloride-based, 80°C–140°C, 1–2 h	Efficient separation and recovery of lignin, High yield of fermentable sugars

### 4.1 Hydrothermal pretreatment

Hydrothermal pretreatment is a widely used, cost-effective, and environmentally friendly method for processing lignocellulosic biomass (Singh et al., 2023). It separates biomass into solid and liquid fractions, with the solid phase primarily containing cellulose and lignin for saccharification and fermentation and the liquid phase containing dissolved hemicellulose. This process enhances the accessibility of cellulose for enzymatic hydrolysis and increases the profitability of biorefining by efficiently utilizing both fractions. Operating at temperatures between 160°C and 270°C and pressures over 5 MPa, hydrothermal pretreatment leverages auto-hydrolysis, where water ionizes to produce hydronium ions that facilitate selective hydrolysis of ether bonds in lignin and acetyl group in hemicellulose decomposition (Shen et al., 2016), lead to polysaccharide and lignin depolymerization. This method significantly improves enzymatic hydrolysis efficiency, and supports the overall economic viability of biorefinery processes (Yang et al., 2018).

Yuan et al. (2019) conducted a short-term two-step hydrothermal pretreatment on tobacco stalks, effectively removing non-structural carbohydrates (mainly hemicellulose) and dissolving them in water. Initially, at a lower temperature of 144°C for 21 min, 11.8 wt% of sugars (based on dry biomass weight) were dissolved. The temperature was then increased to 200°C and maintained for 0 min, resulting in the extraction of 1.56 wt% of high-molecular-weight hemicellulose and 3.95 wt% of low-molecular-weight hemicellulose oligosaccharides, removing 86.98% of non-structural polysaccharides. The residue was primarily composed of cellulose and lignin. Additionally, the study found that after hydrothermal treatment, the residue surface changed from highly ordered to rough, with lignin forming spherical deposits on the surface.

**FIGURE 4:**
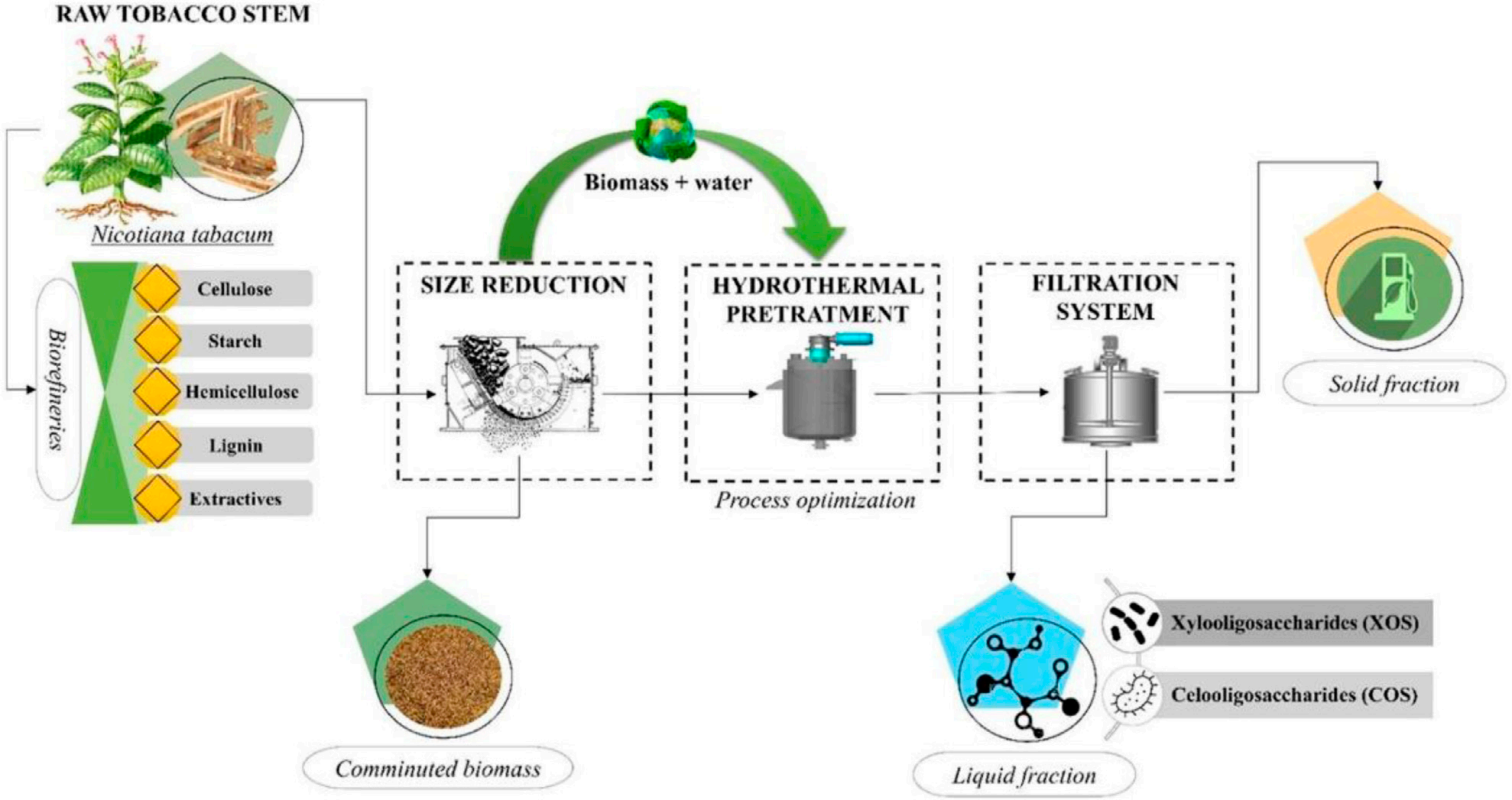
Hydrothermal pretreatment of tobacco stem to produce oligosaccharides (Santana et al., 2023).

Furthermore, Santana et al. (2023) increased the solid content to 17% and performed hydrothermal pretreatment at 190°C for 10 min, selectively converting hemicellulose in tobacco stalks into prebiotic oligosaccharides, achieving a maximum yield of 132 g of oligosaccharides per kilogram of tobacco stalks (Figure 4). These prebiotics can be used in food formulations, dietary supplements, and even pharmaceuticals to help restore gut microbiota. This study highlights the potential of tobacco-derived biomolecules, reinforcing the concept that “tobacco goes far beyond cigarettes,” a concept known for its significant health impacts on millions. Building on this, hydrothermal pretreatment at a higher solid content (20%) and at 190°C for 8 min was found to be the most effective, yielding 49.54% of xylooligosaccharides with a concentration of 11.11 g/L (Santana et al., 2024). Additionally, the residue could be further bioconverted to produce succinic acid with a concentration of 16.88 g/L and a yield of 42%. The entire process can produce 56 kg of oligosaccharides and 78 kg of succinic acid per 1,000 kg of tobacco. This demonstrates the feasibility of using tobacco stalks as valuable raw materials for producing oligosaccharides and succinic acid, laying the foundation for future optimization in clean and sustainable industrial applications. Although lignin removal at the macroscopic level was minimal during hydrothermal treatment, lignin degradation occurred due to extensive cleavage of ether bonds, with its molecular weight drastically reduced from 5,160 to 2,910 g/mol (Sun et al., 2019). This degradation and subsequent condensation caused the lignin to form spherical deposits on the biomass surface upon cooling, which hinders subsequent enzymatic hydrolysis of cellulose. Recently, Li et al. (2024) introduced rhamnolipid surfactants into the hydrothermal pretreatment process, achieving efficient conversion of tobacco stalks into reducing sugars with a concentration of 11.86 g/L, and the residue produced 611.3 mL of hydrogen through enzymatic hydrolysis and fermentation.

Hydrothermal pretreatment is a green and efficient method, but relying solely on temperature increase to enhance the concentration of hydrogen ions and activate biomass components may not achieve optimal results. Utilizing hydrothermal pretreatment as a solvent and incorporating acid, alkali, or metal catalysts to lower the pretreatment temperature and enhance conversion efficiency could be the future direction of hydrothermal pretreatment development.

### 4.2 Steam explosion pretreatment

Steam explosion pretreatment is a widely adopted method for lignocellulosic biomass processing (Hoang et al., 2023). This technique employs high-temperature and high-pressure steam to alter the biomass structure, enhancing enzymatic hydrolysis efficiency. Conducted at temperatures between 160°C and 240°C and pressures of 0.7–4.8 MPa for durations from seconds to min, the process involves rapid depressurization post-steam treatment, causing explosive decompression (Wang et al., 2015). This primarily hydrolyzes hemicellulose, improving cellulose accessibility for enzymatic hydrolysis and increasing the enzymatic hydrolysis rate. The acetyl group hydrolysis in hemicellulose releases acetic acid, which further catalyzes hemicellulose hydrolysis, converting it into fermentable glucose and xylose monomers. Key advantages of steam explosion include high sugar recovery efficiency, cost-effectiveness, and environmental benefits. However, challenges such as potential fermentation inhibitors, stringent pretreatment conditions, and high energy consumption exist (Yu et al., 2022). Despite these, steam explosion remains prevalent in industrial applications requiring high sugar recovery.

**FIGURE 5:**
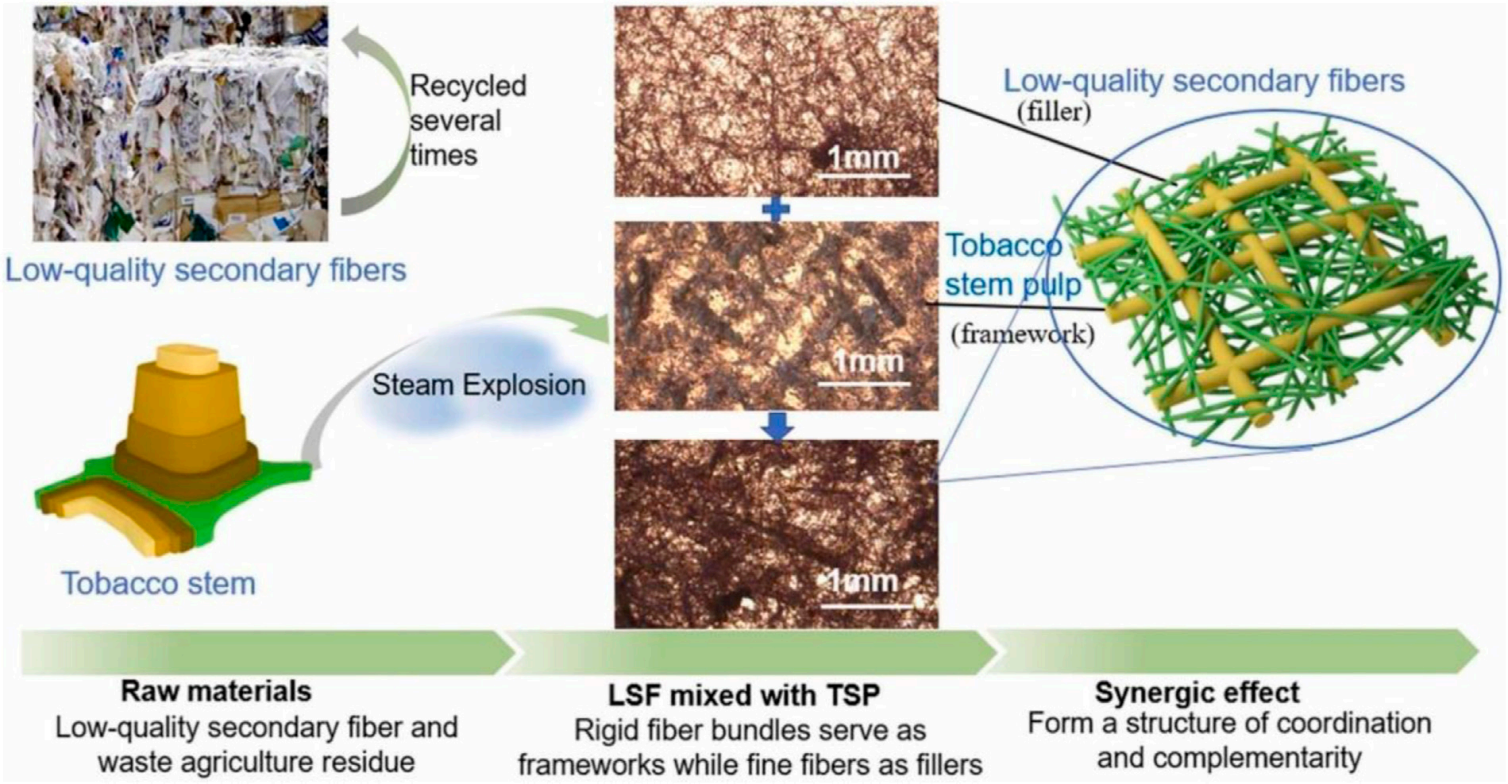
Steam explosion pretreatment of tobacco stem to produce mechanical pulp (Wang et al., 2024).

Han et al. (2015) conducted steam explosion pretreatment of tobacco stems at 0.5 MPa for 20 S. This treatment rendered the tobacco stems loose, facilitating polysaccharide enzymatic hydrolysis. Compared to untreated tobacco stems, the enzymatic hydrolysis efficiency of steam-exploded tobacco stems increased by 231%. Martin et al. (2008), Martin et al. (2002) further investigated steam explosion pretreatment at different durations and found that longer pretreatment times resulted in higher sugar concentrations in the hydrolysate, with relatively low inhibitor concentrations, minimizing the impact on fermentation. The ethanol yield reached 0.39 g/g, outperforming the hydrolysate from sugarcane bagasse. However, nicotine in tobacco stems significantly inhibited enzyme activity and microbial fermentation. Song and Zhang et al. (2021) treated tobacco stems with dilute sulfuric acid followed by steam explosion pretreatment. This method removed up to 85.54% of the nicotine from the tobacco stems and improved the efficiency of carbohydrate enzymatic hydrolysis and ethanol fermentation by 103.36% compared to steam explosion pretreatment alone. In a 5 L fermentation setup, the ethanol concentration reached a maximum of 23.53 g/L, with a glucose-to-ethanol conversion yield of 71.40%. Besides enzymatic hydrolysis for glucose and ethanol fermentation, the steam-exploded tobacco stem residue, due to its larger surface area and higher dissociation degree, can be used for pulping and nanocellulose production. Moreover, Tuzzin et al. (2016) introduced an alkaline activation step in the steam explosion pretreatment process. By pretreating at 175°C for 7 min, followed by bleaching, they found that the nanocellulose content in the cellulose from tobacco stems was higher than that in eucalyptus sulfate pulp, with no significant chemical differences between the two. Incorporating alkali-treated steam-exploded tobacco stem pulp as “backbone” fibers into low-quality secondary fiber paper pulp upgraded the paper from substandard to premium grade (Grade A), significantly increasing the breaking length (64.79%–90.14%) and ring crush index (34.36%–162.96%) (Figure 5) (Wang et al., 2024).

Overall, steam explosion pretreatment is a green and environmentally friendly technology that can significantly enhance the structural looseness of tobacco stem biomass in a short period, facilitating subsequent utilization. However, the process involves high pressure and high temperature, necessitating pressure-resistant equipment and high energy consumption. Future research should focus on optimizing steam explosion pretreatment conditions to reduce energy consumption, improve processing efficiency, and enhance the feasibility of industrial applications.

### 4.3 Acid pretreatment

Acidic reagents have demonstrated the ability to break glycosidic bonds in lignocellulose, effectively dissolving cellulose, hemicellulose, and small amounts of lignin, thereby promoting the release of fermentable sugars (Lv et al., 2024). Compared to hydrothermal pretreatment, acid pretreatment is more commonly used and effective for degrading hemicellulose (and partially lignin) and disrupting amorphous cellulose (Jiang et al., 2023). The primary mechanism involves disrupting interactions between lignocellulosic components, such as van der Waals forces, hydrogen bonds, and covalent bonds, resulting in the dissolution of hemicellulose and the disruption of cellulose and lignin structures. This makes acid pretreatment more effective in lowering pretreatment temperatures and reducing energy consumption in biomass refining compared to hydrothermal methods. Due to the corrosive nature of acids, diluted acids are typically used to reduce equipment corrosion and minimize environmental harm. Although acid pretreatment enhances biomass degradation more effectively than hydrothermal pretreatment, it also generates inhibitors (such as furfural, 5-hydroxymethylfurfural, levulinic acid, and lactic acid), which can impede subsequent enzymatic hydrolysis and fermentation (Lv et al., 2024). Therefore, optimizing acid pretreatment involves balancing its high degradation efficiency with the generation of inhibitors to maximize overall biomass conversion efficiency.

The acid pretreatment of tobacco stems generally employs dilute acids, primarily to remove hemicellulose and enhance the accessibility of cellulose to enzymes. For example, after pretreatment with dilute sulfuric acid at 121°C for 90 min, the total reducing sugar in the hydrolysate reached 20.3 g/L, mainly consisting of hemicellulose-based monosaccharides (Schneider et al., 2017). The residue’s enzymatic hydrolysis yielded 38.1% glucose, and subsequent fermentation produced 0.19 g ethanol per gram of raw material. Besides sulfuric acid, other inorganic acids, such as HCl and HNO_3_, have also been studied (Temitayo et al., 2019). However, their efficiency is lower than that of sulfuric acid, possibly because H_2_SO_4_ being a diprotic acid, can continuously provide hydrogen ions, whereas HCl and HNO_3_ are monoprotic and volatile acids, making them less effective for tobacco stem pretreatment. Using a weaker organic polyacid like citric acid for the pretreatment of tobacco stems has shown higher efficiency than sulfuric acid. This may be because citric acid, being a weak polyacid, can continuously supply hydrogen ions to promote acid pretreatment, while its mild acidity can reduce the condensation of lignin structures in the biomass, thus decreasing inhibition of cellulose accessibility to enzymes. The significant advantage of acid pretreatment of tobacco stems is the removal of toxic substances such as nicotine, ensuring safe and high-value utilization in subsequent processes (Zhang et al., 2021). Studies have shown that dilute acid pretreatment can remove up to 85.54% of nicotine while enhancing enzymatic hydrolysis. Although dilute acid pretreatment can promote the acid hydrolysis of hemicellulose to produce oligosaccharides and even monosaccharides like xylose, it can also lead to side reactions that produce furfural and fatty acids and promote partial degradation of lignin to phenolic compounds, which inhibit subsequent enzymatic hydrolysis and fermentation (Li et al., 2023). To improve ethanol fermentation yield, tobacco stems subjected to dilute acid pretreatment can undergo further mild alkaline pretreatment, achieving the removal of 47% hemicellulose and 65% lignin (Gong et al., 2023). After enzymatic hydrolysis, the residues can then be co-fermented, producing 0.19 L ethanol per kilogram of tobacco stems. In summary, acid pretreatment is an efficient method for processing tobacco stems. By optimizing the types of acids and pretreatment conditions, it is possible to further enhance biomass conversion efficiency and reduce the generation of inhibitors, laying the foundation for industrial application.

### 4.4 Alkaline pretreatment

Different from hydrothermal and acid pretreatment, alkaline pretreatment can effectively dissolve lignin (Xu et al., 2016; Ntakirutimana et al., 2022). Through saponification and solvent reactions, the bonds in lignin-carbohydrate complexes (LCC), such as ester bonds, aryl ether bonds, alkyl-aryl bonds, and glycosidic bonds, are broken (Dong et al., 2019). Subsequently, acetyl and uronic acid groups in hemicellulose are also removed (Xu et al., 2016). These reactions lead to structural changes and degradation of lignocellulosic biomass, increasing the exposure of reactive sites and promoting cellulose swelling and partial decrystallization, thereby facilitating further enzymatic hydrolysis (Kim et al., 2016). Additionally, tobacco stalk biomass contains ash components like silica, which exist as silica deposits and protect the cell wall from digestibility (Khaleghian et al., 2017). Alkaline pretreatment can dissolve these silica compounds into the solution as silicates, promoting the breakdown and utilization of cell wall components. Generally, unless extensive delignification is implemented, alkaline pretreatment is less effective in removing the recalcitrance of plant biomass. However, it has the advantage of eliminating fermentation inhibitors (Jönsson and Martín, 2016). Nevertheless, the recovery of alkaline substances requires expensive capital investment and remains a significant barrier to alkaline pretreatment.

**FIGURE 6:**
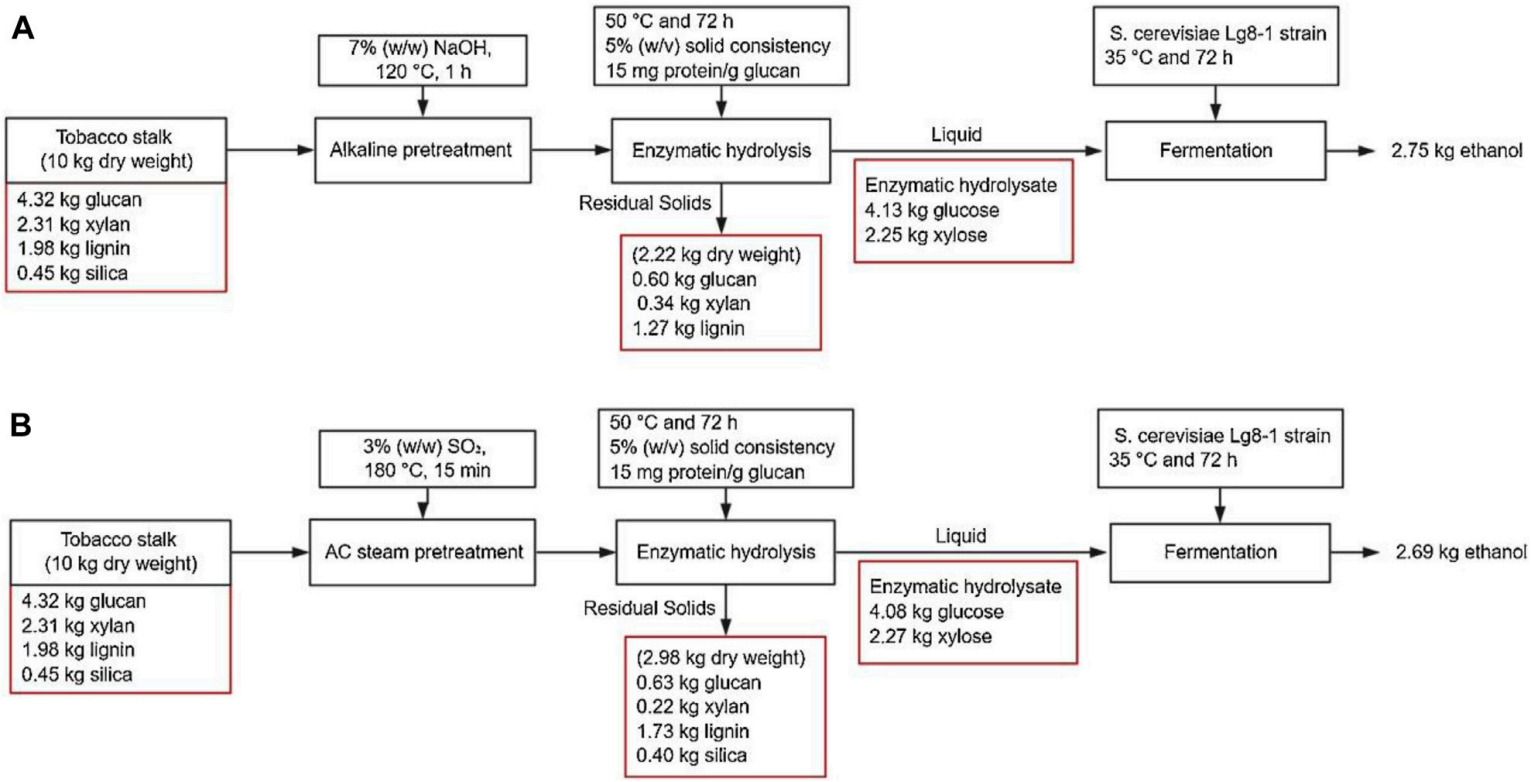
Mass balance flow diagram on a 10 kg basis of tobacco stalk for two pretreatments: **(A)** alkaline, **(B)** acid-catalyzed steam pretreatments, enzymatic hydrolysis, and ethanol fermentation (Yuan et al., 2019).

Alkali pretreatment of tobacco stalks not only mitigates the formation of fermentation inhibitors but also promotes the solubilization of lignin, thus enhancing the enzymatic hydrolysis efficiency of carbohydrates (Figure 6) (Yuan et al., 2019; Guo et al., 2021). The pretreatment conditions for tobacco stalks are relatively mild compared to other biomass types. For instance, sodium hydroxide pretreatment at 90°C has been shown to increase enzymatic hydrolysis efficiency by 165.7%, with subsequent simultaneous saccharification and fermentation yielding a 138.0% increase in ethanol production (Guo et al., 2021). Moreover, CaO has also been used effectively for the alkali pretreatment of tobacco stalks (Sophanodorn et al., 2020a; Sophanodorn et al., 2020b). This method resulted in a 79.35% monosaccharide yield upon enzymatic hydrolysis (Sophanodorn et al., 2020b). During alkali pretreatment, components such as lignin, hemicellulose, extractives, and a portion of amorphous cellulose are partially removed, enhancing the thermal stability of the residue but lowering the activation energy (Dallé et al., 2021). Additionally, the crystalline cellulose in the residue transforms from cellulose I to cellulose II, with the residue surface becoming rougher due to the removal of lignin and hemicellulose, making it suitable for applications as reinforcing fillers in composite materials. Despite the effectiveness of alkali pretreatment in lignin removal, hemicellulose remains in the residue. To address this, a sequential hydrothermal-alkali pretreatment can be employed to separate cellulose, hemicellulose, and lignin from tobacco stalks (Sophanodorn et al., 2020c; Wang et al., 2022). This combined pretreatment method significantly enhances enzymatic hydrolysis and fermentation efficiency, achieving ethanol yields of up to 75.74 g/L. During the hydrothermal-alkali process, the bonds between lignin and carbohydrates are cleaved, resulting in lignin with low carbon content and slightly increased molecular weight due to the cleavage of aryl-ether bonds and partial condensation during hydrothermal treatment (Liu et al., 2021). Interestingly, the regenerated lignin exhibits regular nanoparticle morphology and excellent UV absorption properties, making it suitable for lignin-based UV shielding materials. Furthermore, incorporating oxidants during alkali pretreatment improves the efficiency of tobacco stalk processing, yielding residues with 92.41% cellulose purity (Martin et al., 2008; Zhan et al., 2024). The cellulose crystallinity changes and its thermal stability increases from 261°C to 369°C. This high-purity cellulose can be used to produce food preservation films using plasticizers such as glycerol and sorbitol through solution casting, achieving high-value utilization of cellulose (Qian et al., 2023).

Overall, alkali pretreatment operates at relatively mild temperatures and effectively removes lignin, enhancing the accessibility of carbohydrates to enzymes. The transformation of cellulose crystalline structure is advantageous for producing cellulose-based materials. Compared to acid pretreatment, alkali pretreatment effectively eliminates inhibitors, facilitating subsequent enzymatic hydrolysis and fermentation. Therefore, alkali pretreatment is a prevalent method for processing tobacco stalks, demonstrating significant potential for industrial applications.

### 4.5 Organosolv pretreatment

Compared to hydrothermal, acid, and alkaline pretreatment methods, organosolv pretreatment is more effective at dissolving lignin, efficiently removing it from the biomass, and dissolving it in the solvent (Ferreira and Taherzadeh, 2020). This process also penetrates and breaks down the internal chemical bonds of cellulose and hemicellulose. During this process, the dissolution of hemicellulose and delignification enhance the pore volume and surface area of cellulose, improving its availability for saccharification and enzymatic hydrolysis (Xu et al., 2023). Since the biomass components are highly polar substances rich in hydroxyl groups, low-boiling alcohols (such as ethanol and methanol) and high-boiling alcohols (such as glycerol, ethylene glycol, and tetrahydrofurfuryl alcohol) are commonly used in organosolv pretreatment (Sun et al., 2022; Sun et al., 2023). However, due to the large molecular weight of biomass, its solubility is relatively poor. To achieve more effective pretreatment, acid or alkaline catalysts are added to accelerate the hydrolysis of aryl ether bonds in lignin and hemicellulose, allowing them to dissolve in the organic solvent. Despite the effectiveness of organosolv in dissociating biomass, most organic solvents are costly, increasing the overall cost of the pretreatment process (Ferreira and Taherzadeh, 2020). Additionally, the flammability and volatility of organic solvents are significant drawbacks that must be considered in organosolv pretreatment.

Currently, research on the organic solvent pretreatment of tobacco stems is relatively limited. This is primarily due to the higher cost of organic solvents compared to hydrothermal treatments, which hinders industrial promotion. Additionally, the presence of nicotine and solanesol in tobacco stems affects the efficiency of the pretreatment, requiring additional extraction steps and further increasing costs. Studies have shown that using ethylene glycol as an organic solvent to pretreat tobacco stems results in a lignin removal rate of only 13.9%, significantly lower than the delignification rates for wheat straw and corncobs. This is mainly because solanesol in tobacco stems weakens the strong hydrogen bond interactions between free hydroxyl groups in lignin and ethylene glycol. To mitigate the influence of solanesol, Chen et al. (2022) used n-hexane extraction to remove solanesol before ethylene glycol pretreatment, successfully increasing the lignin removal rate to 40.5%, an improvement of 191%. This method provides an effective solution for the organic solvent pretreatment of tobacco stems.

Despite the potential of organic solvent pretreatment in addressing lignin removal in tobacco stems, further research and optimization are needed for large-scale industrial applications. This includes finding more cost-effective solvents and improving pretreatment processes to reduce costs and enhance efficiency.

### 4.6 Ionic liquids pretreatment and deep eutectic solvents pretreatment

According to the principles of green chemistry, environmentally friendly solvents are crucial for sustainable processes (Roy et al., 2020). Industrial solvents often contain toxic substances, driving the demand for green alternatives like ionic liquids (ILs) and deep eutectic solvents (DES) (Roy et al., 2020). ILs, particularly imidazole-based ones, excel in dissolving cellulose, hemicellulose, and lignin, aiding enzymatic hydrolysis and biomass conversion into fermentable sugars and biofuels (Achinivu et al., 2024; Hernandez et al., 2024). They can be recycled due to their lack of vapor pressure, but their high cost and toxicity to cellulases limit large-scale application. Residues of lignin and hemicellulose in ILs can increase viscosity, complicating further processing. DES, derived from natural, non-toxic, and inexpensive resources, offers a promising alternative to ILs (Hong et al., 2020). They are compatible with enzymes, cost-effective, and simple to synthesize without complex purification. DES forms competitive hydrogen bonds with carbohydrates and hydroxyl groups in lignin, hydrolyzing LCC and breaking ether bonds between lignin and hemicellulose (Shen et al., 2019a; Wang et al., 2019). DES selectively dissolves lignin, enhancing biomass deconstruction and high-value utilization (Shen et al., 2019b; Wang et al., 2020). Despite these benefits, both ILs and DES face challenges with increased viscosity due to residual lignin and hemicellulose, complicating subsequent processing and applications.

IL pretreatment and DES pretreatment, as relatively green and advanced technologies, have been applied to tobacco stalks to a lesser extent, mainly focusing on lignin separation and characterization, interpreting structural changes of lignin during pretreatment, and identifying its unique properties. Similar to other biomass, imidazolium-based ionic liquids exhibit high solubility for tobacco stalk lignin, especially 1-ethyl-3-methylimidazolium diethylphosphate ([Emim][DEP]), which has the highest solubility due to its strong acidity and hydrogen bonding capability (Fan et al., 2018). After [Emim][DEP] pretreatment, the removal of lignin renders the surface of tobacco stalks rough, facilitating subsequent enzymatic hydrolysis and fermentation. The purity of separated lignin exceeds 85%, with extraction rates reaching up to 90.21%. As reaction conditions become more severe, the regeneration rate of lignin decreases, likely due to lignin degradation during IL pretreatment, resulting in lignin fragments that cannot precipitate and regenerate. At higher temperatures, lignin further undergoes condensation.

**FIGURE 7:**
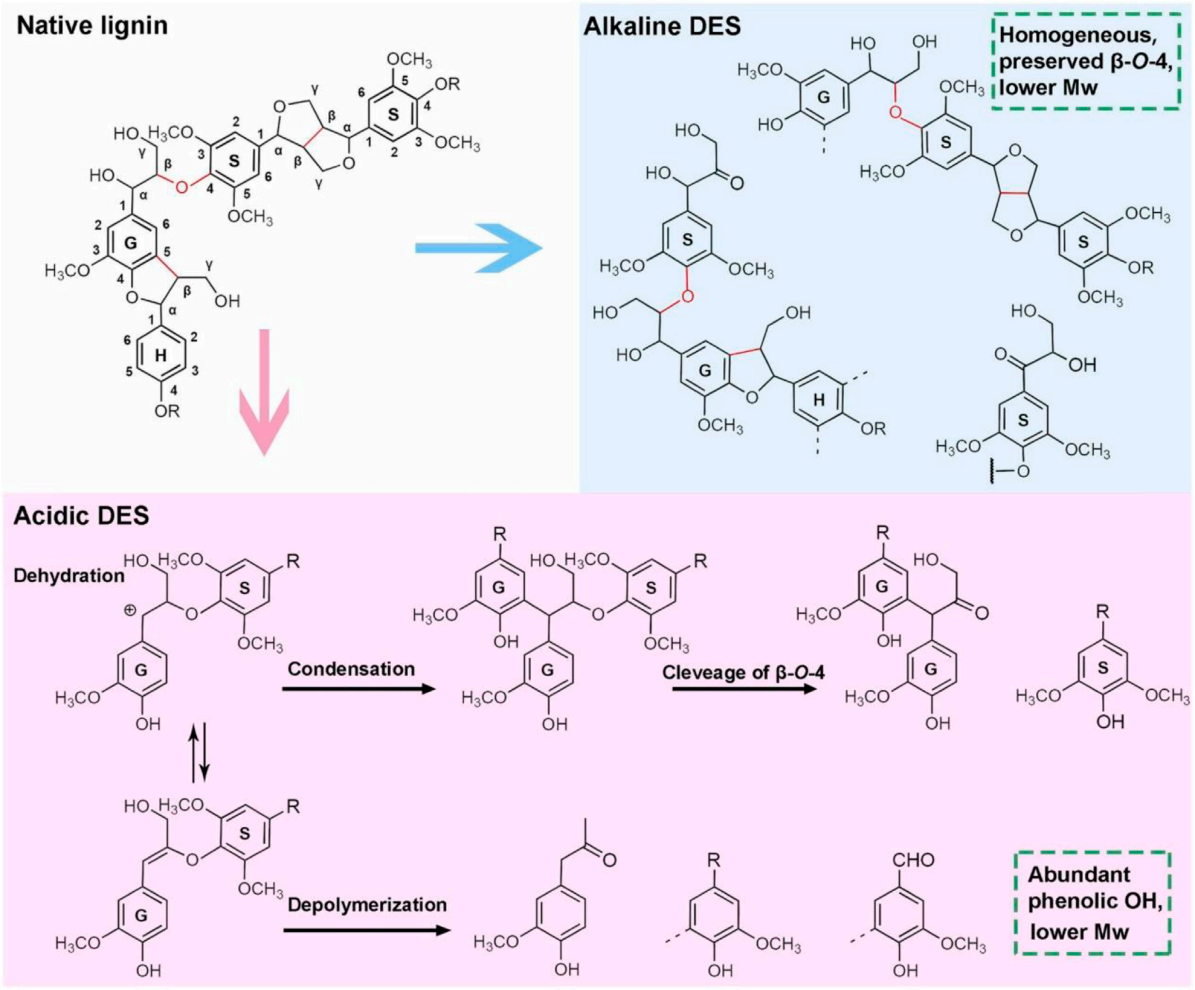
Potential mechanism of lignin in tobacco stalk macromolecule in acidic/alkaline DESs (Wang et al., 2022).

DES also exhibits strong solubility for lignin in tobacco biomass (Wang et al., 2022; Wang et al., 2022). Studies on the pretreatment of tobacco stalk lignin using acidic, neutral, and basic DES have shown that acidic DES (e.g., lactic acid/choline chloride) can efficiently separate and extract tobacco stalk lignin. This is likely because acidic DES not only has high solubility for lignin but also efficiently cleaves hemicellulose, promoting lignin separation. Conversely, basic DES (monoethanolamine/choline chloride) and neutral DES (ethylene glycol/choline chloride) show less significant effects on hemicellulose degradation, and the dispersion of lignin decreases, resulting in lignin with a uniform molecular weight. However, lignin in acidic DES undergoes significant degradation and condensation, whereas basic DES pretreatment maintains the lignin aryl ether bond content (Figure 7). Therefore, for DES pretreatment of tobacco stalks, if the goal is to improve lignin separation efficiency without focusing on its structural changes, an acidic DES system should be used. If the objective is to obtain structurally intact lignin for subsequent use, basic DES solvents should be employed.

Although the application of ionic liquid pretreatment and DES pretreatment in tobacco stalks is currently limited, their green solvents, zero vapor pressure, high safety, and recyclability significantly promote the green and efficient pretreatment of tobacco stalks.

## 5 Issues and prospects of tobacco stalk pretreatment

The pretreatment of lignocellulosic biomass has been crucial for bioenergy production for over a century (Zhao et al., 2022). Overcoming the recalcitrance of lignocellulosic materials through pretreatment is essential for producing various valuable products. However, unlike other biomass types, tobacco stalks contain toxic substances such as solanesol and nicotine, which render common biomass pretreatment technologies less effective. Therefore, this paper reviews the latest advances in the pretreatment of tobacco stalk lignocellulosic biomass, discussing the technological challenges and future opportunities. Currently, the pretreatment of tobacco stalk lignocellulosic biomass remains in the experimental stage and faces several issues.

(1) Key Points of Different Pretreatment Methods: Different pretreatment methods focus on breaking down the biomass’s resistance to degradation and utilizing specific components, often leading to the wastage of other components. For instance, acid pretreatment primarily degrades hemicellulose, while lignin may undergo severe condensation, making it difficult to utilize further. Alkaline pretreatment effectively extracts lignin but may degrade carbohydrates, resulting in sugar loss. Although IL and DES pretreatments efficiently deconstruct biomass, hemicellulose and lignin cannot fully precipitate and regenerate, complicating solvent recycling.
(2) Cost and Waste Liquid Treatment: Current pretreatment technologies mainly focus on efficiently deconstructing tobacco stalks and their high-value utilization. However, industrial applications require attention to the overall process cost and the minimization of the discharge of waste liquids to achieve green economic development in the high-value utilization of tobacco stalk biomass. High energy consumption and costs in the pretreatment process limit its industrial application. Waste liquid treatment also needs to be addressed to ensure the environmental and economic feasibility of the process.
(3) Utilization of Other Valuable Components: Although tobacco stalk biomass mainly contains cellulose, hemicellulose, and lignin, it also includes substances like nicotine and solanesol. Despite being harmful, these substances have high utilization value in other fields, such as solanesol being an important pharmaceutical intermediate. Current pretreatment often ignores the extraction of solanesol, focusing only on the separation of the three main components. Therefore, developing new technologies that can efficiently separate the main components and extract valuable by-products is necessary.

Through the measures below, efficient pretreatment and high-value utilization of tobacco stalk biomass can be achieved, promoting its application in bioenergy and bioproduct production and contributing to sustainable development.

(1) Coupling Multiple Pretreatment Methods: Different pretreatment technologies have their own advantages and disadvantages and should be combined for efficient biomass utilization. For example, using organic solvents to extract solanesol and nicotine, hydrothermal pretreatment to remove hemicellulose, and alkaline or DES pretreatment to remove lignin, thus efficiently deconstructing tobacco stalk biomass and laying the foundation for subsequent high-value utilization.
(2) Life Cycle Assessment and Techno-Economic Analysis: Conduct life cycle assessments and techno-economic analyses of existing pretreatment technologies to consider the overall process’s economic and sustainability aspects. This includes researching how to economically and efficiently treat the waste liquids generated from pretreatment and even further utilizing them for high-value applications. This will help identify the most economically and environmentally feasible pretreatment methods and promote the industrial application of tobacco stalk pretreatment technologies.
(3) Developing High-Quality Products: Develop more technologies targeting tobacco stalk biomass to produce high-quality products and broaden their application range. In addition to common cellulose, hemicellulose, and lignin, explore the high-value utilization of by-products like nicotine and solanesol. For instance, solanesol, as a pharmaceutical intermediate, has a broad market prospect. Through reasonable pretreatment and separation technologies, its extraction efficiency and purity can be improved, thereby increasing the overall economic value of tobacco stalk biomass.
(4) Technological Innovation and Optimization: Continuously innovate and optimize existing pretreatment technologies to improve the utilization efficiency of tobacco stalk biomass. For example, combining genetic engineering techniques to optimize microbial strains and enhance biodegradation efficiency, adopting advanced reactor configurations and visualization technologies to optimize the control parameters of the pretreatment process; using novel catalysts and reaction media to improve pretreatment selectivity and efficiency. These technological innovations will provide new ideas and methods for the high-value utilization of tobacco stalk biomass.
